# Editorial Notes

**Published:** 1911-12

**Authors:** 


### Editorial Notes.

**Medical Education and the Board of Education.**

THE Government has published a Blue Book. Which after all is what Governments are for. And some Blue Books make very good reading, almost every taste being catered for — history, geography, agriculture, science and fiction—the taxpayer pays his money, and the reader may take his choice.

The Board of Education has made a fat book bound in blue about " Universities and University Colleges receiving Parliamentary Grants," wherein are many admirable facts and figures relating to the cost of University education and how it is met. The figures have a simple air of truth about them that deters us from any destructive criticism. We will leave them with the Baconian maxim that " a pleasing figure is a perpetual letter of encouragement," and pass on to the Preface.

Mr. Runciman, as President of the Board of Education, has figuratively blessed the book with an introduction from his own ministerial pen, and no doubt his words are too sacred for correction by any of the officials in his department. So he has given expression to some very original research into the history of medical education. It is his ambition to see medical education directed and controlled by the Board of Education. The unparalleled success of the Board in the management of elementary education in this country will inevitably secure a rapturous acceptance for the fatherly guidance of the Board in medical education.

But we do not like this particular Danäid with his own particular contribution to the history of the evolution of English medical schools. He almost laments the fact that five general medical schools still exist so blind as to forego the advantages of a Government Grant coupled with Government supervision. Parenthetically, there rises to the mind a recollection of the University of Oxford perversely informing the General Medical Council some few years since that the suggestions of the Council for improving the medical course at Oxford were declined with thanks, yet Oxford still lives.

The Blue Book confesses to a difficulty in dealing with the London hospitals, for there the medical school, unlike those of the provinces, has frequently been an offshoot of the hospital. Apparently there is a feeling that where a hospital gave rise to its own medical school the Government will find some difficulty in interfering to the full extent of its desires with the management of the medical school. We anticipate that these forebodings will prove true.

It is in the highest degree likely that the London hospitals will continue to appoint to their staff physicians and surgeons whose attainments qualify them to practise to the best advantage among the sick who claim their help, and from these practisers of the art students will continue best to learn their art.

We are apprehensive lest Board of Education control of medical education should mean the intruding of mere teachers for masters in the domain of medicine and surgery. It is the masters of medicine in the old sense of whom medicine to-day stands most in need. And our observations of the hand of the Board as evidenced by its system of inspection in other branches of learning do not encourage the idea that the ways of Hippocrates and Sydenham, or the manly intellectual culture, the "fine confused feeding" of Arbuthnot, Heberden and Gregory would lead any other than an exiguous existence in medical education. But the President of the Board of Education makes a reluctant admission that medical schools originating in hospitals do not stand quite on all fours with medical schools which, like Topsy, "just growed," or were born of some pre-existing University or College. The meaning of the remark is cryptic, but there is an inaccuracy in his assumption that the majority of provincial medical schools do not trace their origin to some hospital.

Bristol, at any rate, is in precisely the same position as most of the London hospitals, and the medical school in Bristol antedates many of those in London, but with this difference, that from the Royal Infirmary and General Hospital sprang the Bristol Medical School which brought into being a University College, and finally in the fulness of time, and by the generosity of large-hearted citizens, a University crowned and cemented the whole edifice.

This the Board of Education will do well to bear in mind, if in its wisdom it decides to direct the whole of the medical education in the United Kingdom. It is by no means certain that the Government will order these affairs any better than the pre-existing organisations. A Government Board may even fail to rival the achievements of Hunter, Baillie, Syme and Paget, to name but a few of those who have lent lustre to the honourable roll of masters in medicine.

In their efforts to improve by inspection the teaching ot medicine they will do well to remember Latham's advice not to "rebuke the experience of all past times and dictate to the experience of all future," lest in their praiseworthy desire to mould the teaching of medicine to a Government ideal they discover the truth of Bernard Shaw's gibe: "He who can does, he who cannot—teaches!"

**The Compulsory Notification of Pulmonary Tuberculosis.**

THE following notes on this important subject have been supplied by Dr. D. S. Davies, Medical Officer of Health:—

The Public Health (Tuberculosis) Regulations, 1911, enforcing the compulsory notification of Pulmonary Tuberculosis by all Medical Practitioners and School Medical Inspectors, do not come as a surprise, but rather as the necessary and promised complement to the compulsory Regulations of 1908 and 1911 in regard to Poor Law and Hospital cases.

Ten years ago any attempt at compulsory notification of Pulmonary Tuberculosis in England would have met with considerable opposition and resentment, for it was rightly felt that the disabilities which necessarily apply tc persons suffering from the ordinary eruptive fevers would involve intolerable hardships to many phthisical patients.

The experience gained, however, with a system of compulsory notification since 1903, under a private Act in Sheffield, under similar powers in Bolton, Burnley, and Oldham, and under a voluntary system in other towns, including Bristol, has shown that, in place of acting to the detriment of the patient, there is abundant evidence, to use the words of the Chief Medical Officer of the Local Government Board, "of notification bringing within reach of the patient help that had previously been unobtainable." Special care is also taken in all the recent Regulations to safeguard the interests of the patient, and to ensure the privacy of all notifications made thereunder.

The Regulations come into operation on the 1st January, 1912, and do not supersede but supplement, and in some respects extend, the Poor Law and Hospital Regulations. For example, Article IX. (1) is wider than the corresponding Article in previous Regulations, in so far as it includes a power to provide medical assistance.

The powers and duties of Councils are pointed out in the Memorandum accompanying the Order to include :—

> The provision of Sanatoria or the power to contract for their use. The provision of Dispensaries or Out-patient Hospitals, or the power to contract for their use. The power to supply, on the advice of the Medical Officer of Health, such medical assistance, facilities and articles as may be necessary for detecting Pulmonary Tuberculosis, for preventing the spread of infection, and for removing conditions favourable to infection. The power to appoint any necessary additional officers.

Local Authorities are also advised to carefully take account of local agencies, and to make full use of their powers of preventing or removing conditions likely to prove injurious to health.

Article IV. (1) relieves a Medical Practitioner of the duty of notifying a case which has to his knowledge already been notified, and it is pointed out in the Memorandum that a physician called in for consultation would not, in general, be responsible for notification, and that notification would seldom be required from the Medical Officer of a Sanatorium or Convalescent Home for Consumptives. The Regulations do not apply (Article X.) to Medical Officers of a Poor Law Institution, or District Medical Officers, to Medical Officers of Hospitals, Prisons, Certified Reformatory Schools or Institutions for Lunatics, or to Medical Examiners of candidates for some office or employment, of passengers and crews of emigrant ships, or on behalf of an insurance company of a person proposing to insure his life at the risk of that company. Inmates of Crown buildings or ships are also exempted from the operation of the Regulations.

Notification is to be made by Medical Practitioners or School Medical Officers in every case coming within the Order within forty-eight hours after first becoming aware that a patient or a child attending a Public Elementary School is suffering from Pulmonary Tuberculosis. The fee for each notification is 2s. 6d., which covers all expenses, including the cost of transmission, except that no fee shall be payable to a School Medical Inspector.

The Regulations are made under Section 130, Public Health Act, 1875, as amended by the Public Health Act, 1896, and carry the usual penalty of £50.

Medical Practitioners may find, under the present somewhat strained relations with another Government Department, some balm to their parched lips in the following official pronouncement :—

> "The (Local Government) Board feel sure that the duty which is imposed will be loyally undertaken by a profession which has already contributed so greatly to the furtherance of social efficiency, and which has responded so readily to the demands which have been made from time to time upon its special knowledge and experience."

**Bristol Medical Library.**

The history of the Medical Library (page 335) written by Mr. L. M. Griffiths gives an interesting account of the continuous struggles of the profession in Bristol to get and keep a collection of books and magazines which might serve the needs of the medical men in Bristol and the neighbourhood. The story is one of long and active efforts by succeeding generations of men who have appreciated the value of reading. The continuity and persistence of their labours is remarkable; so too has been the success with which difficulties have been overcome. Frequent changes of premises and insecurity of tenure have interfered little with the steady growth of the Medical Library, but at every crisis that has arisen there have not been wanting men who, all dedicated to closeness and the bettering of their minds, pursued their self-appointed task with zeal. Thus each change has been but a further stage of growth, the resources of the Library in funds and books have multiplied and are still increasing. It is the great and widely-prized possession not of a few mere book-worms, but of a large circle of busy doctors active in their practice and eager to avail themselves of the knowledge of other brains and other countries than their own. It is one of the greatest advantages that can be thought of, as Dr. Dell[1] wrote long ago, to "spend some part of the day in learning or study, and the other part of the day in some lawful calling; or one day in study, and another in business, as necessity or occasion shall require."

Mr. Griffiths's paper describes how rapidly the Medical Library grew in the first ten years after it came into the hands of the Medico-Chirurgical Society, from 1879 books and 79 periodicals in 1891 to 9371 books and 189 periodicals in 1901.

Meanwhile the advantages derived from the united endeavours of University College and the Society were immense. The profit to the Society cannot be estimated by the mere size

[1] "The Right Reformation of Learning, Schools, and Universities," by William Dell, in his *Select Works*. London: John Kendall. 1673.

of the Library to which its members had access. The old Medical Library became the active centre of medical life in Bristol. It was regarded as the head-quarters not only of the Medical School, but also of the Society and of the British Medical Association. It has played no small part in establishing that unity in the medical profession which at the present juncture has been made manifest. To our librarians, Mr. L. M. Griffiths (1890–1902) and Mr. C. K. Rudge, who now occupies the post, our grateful thanks are due for their honorary services and valuable work. There is one important development to which Mr. Griffiths's paper does not refer, namely the establishment of a lending department in 1908. Previously members could only consult the books in the Library, but in 1908 Mr. Rudge and the Library Committee drew up a scheme by which volumes might be borrowed by members. This was done, moreover, without any increase of subscription or additional charge, for the extra cost has been more than compensated by the increased membership of the Society which resulted from this useful privilege.

But this year another critical change has taken place, a change unwelcome perhaps to many of us.

The University authorities, sensible of the need for adequate administrative premises, informed the Society that the Medical Library must be moved to make room for a Council Chamber, and gave notice that the agreement with the Society should terminate in 1912. It was within the rights of the Society to cling to the old premises until the expiration of the agreement, but there were other considerations which called for a more generous acquiescence.

The needs of the Medical Department of the University were well known to the Society, proper educational equipment could not be provided in the old buildings, and the Council of the University were able to offer laboratory and other accommodation for the Medical School in the new buildings such as could not have been contemplated in the old. So, not without many regrets for past associations, it was decided to quit the building that had served so well for nearly twenty years in order that the students of to-day might benefit at once by increased accommodation and modern equipment.

The Medical Library now finds itself settling down into the east wing of the old Blind Asylum, where, although there is no single room comparable to the fine chamber it has left, there is ample space for the housing of its books and magazines.

We may look forward to a fresh impulse to the growth of the Library, for in future the contributions of the University to the University section of the Library will be on a scale far beyond the best efforts of University College. Presently the members of the Society will find themselves in joint possession of a Library undreamt of by those who in 1832 made the small beginning in Orchard Street.

We miss just now the large room where formerly the meetings were held, but the University are sparing no pains to place at the disposal of the Society an equally appropriate meeting-place. In the meantime we may comfort ourselves in our discomfort with the adage that " Some falls are means the happier to arise."

